# Effects of Dapagliflozin on Novel Inflammatory Markers in Heart Failure Patients

**DOI:** 10.1155/crp/5537675

**Published:** 2026-02-03

**Authors:** Oktay Senoz, Mustafa Sezen

**Affiliations:** ^1^ Department of Cardiology, Bakırcay University Cigli Training and Research Hospital, Izmir, Turkey

**Keywords:** dapagliflozin, heart failure, SGLT-2 inhibitors, systemic immune–inflammation index, systemic inflammation response index

## Abstract

**Background:**

Sodium–glucose cotransporter‐2 (SGLT‐2) inhibitors have been established to decrease hospitalizations and cardiac death within all heart failure groups. The exact mechanisms by which the oral antidiabetic medication dapagliflozin achieves this advantage are still unknown. The potential beneficial effects of dapagliflozin on inflammation and the immune system may contribute to these mechanisms.

**Method:**

The laboratory and echocardiographic data of 191 consecutive patients who were started on dapagliflozin due to heart failure were compared before and 6 months after the treatment began. The systemic immune–inflammation index (SII) and the systemic inflammation response index (SIRI) were calculated using the following formulae: (platelet × neutrophil)/lymphocyte and (neutrophil × monocyte)/lymphocyte, respectively.

**Results:**

The mean age of the patients included in the study was 66.17 ± 10.7 years. A total of 156 patients (81.7%) had diabetes mellitus. Seventy patients (36.6%) had heart failure with reduced ejection fraction (HFrEF), 31 (16.2%) had heart failure with mildly reduced ejection fraction (HFmrEF), and 90 (47.1%) had heart failure with preserved ejection fraction (HFpEF). While no significant change was observed in echocardiographic parameters with dapagliflozin treatment (*p* > 0.05), a significant decrease was detected in the SII and SIRI (1357.4 ± 1404.3 vs. 805.8 ± 586.7, *p* < 0.001 and 3.68 ± 3.6 vs. 2.19 ± 1.7, *p* < 0.001). In these indices, a consistently significant decrease was observed in all groups, irrespective of the type of heart failure and the presence of diabetes mellitus (*p* < 0.005).

**Conclusion:**

With dapagliflozin treatment, the most recent inflammation parameters, SII and SIRI, have significantly decreased. This effect may be one reason for the cardiovascular benefits of dapagliflozin treatment.

## 1. Introduction

Heart failure is a heterogeneous syndrome, and both its diagnosis and the categorization of patients are challenging. Left ventricular ejection fraction (LVEF) serves as a phenotypic marker to identify underlying pathophysiological mechanisms and guide treatment decisions [1, 2]. Currently, patients with heart failure are classified into three groups: heart failure with reduced ejection fraction (HFrEF; LVEF < 40%), heart failure with mildly reduced ejection fraction (HFmrEF; LVEF 40%–49%), or heart failure with preserved ejection fraction (HFpEF; LVEF ≥ 50%)[3].

It is estimated that approximately 64.3 million people live with heart failure all around the world [4]. In developed countries, the prevalence of heart failure is estimated to be around 1%–2% of the adult population [5, 6]. Despite differences in diagnostic criteria, most studies report that more than half of all heart failure patients in the general population have preserved LVEF, and this rate is expected to increase [7].

Sodium–glucose cotransporter‐2 inhibitors (SGLT2i) promote urinary glucose excretion by avoiding renal reabsorption of filtered glucose to lower blood glucose, which was initially regarded as glucose‐lowering agents for patients with Type 2 diabetes [8–10]. There is also a lower risk of hypoglycemia compared with insulin treatment. The clinical use of SGLT2i has expanded well beyond their glucose‐lowering effects [11]. Recently, dapagliflozin has been associated with a 15% reduction of fatal and nonfatal cardiovascular (CV) disease events compared with insulin treatment [12].

The DAPA–HF and DELIVER trials clearly demonstrated that dapagliflozin is superior to placebo in preventing CV deaths and HF events in both patients with HFrEF and patients with HFpEF [13, 14].

It has been established that SGLT2i reduce adverse cardiac events, but the exact mechanism by which they achieve this effect is not clearly understood. There are several theories regarding their possible positive effects. These include reducing cardiac preload and afterload, improving myocardial energy kinetics, and having positive effects on cardiac remodeling and fibrosis [15, 16]. One reason may be that SGLT2i have pathophysiological roles via sodium and calcium regulation along with cardiac fibrosis and inflammation [17, 18]. Cardiac fibrosis and inflammation are widely considered to be the common pathways involved in heart failure progression, resulting in adverse cardiac structural remodeling that leads to impaired ventricular compliance and cardiac function [19].

The inflammatory response is now recognized as a major contributor to the development of chronic heart failure (CHF); in addition, inadequate organ perfusion caused by inadequate circulating volume and reduced cardiac output are also major causes of CHF.

Prediction of the inflammatory response in heart failure patients may be important for determining prognosis. Previous studies have shown that various metabolic and nutritional inflammatory markers can be used to evaluate the clinical prognosis of patients with advanced heart failure and implantable cardioverter defibrillators (ICDs) [20–22].

Systemic immune–inflammation index (SII) is a good indicator of systemic inflammation, and its association with many CV diseases has been previously demonstrated. Previous studies have identified SII as a new independent predictor of mortality in advanced CHF patients with renal dysfunction [23]. Additionally, this study reported that SII could be used in heart failure monitoring and to assess response to treatment.

In the literature, there is no study demonstrating the positive effects of dapagliflozin on heart failure using the recently introduced inflammation markers, SII and systemic inflammation response index (SIRI).

With this study, we aimed to determine the positive effects of dapagliflozin on heart failure by using the SII and SIRI.

## 2. Method

The study began after obtaining approval from the local ethics committee and informed consent from the included patients.

Between January 2024 and September 2024, 191 consecutive patients either with or without diabetes who were diagnosed with heart failure and started on dapagliflozin treatment were included in the study. Inclusion criteria for the study were as follows: age being over 18 years, presence of HFrEF or HFmrEF or HFpEF, New York Heart Association (NYHA) Class II, III, or IV symptoms, clinical stability after acute decompensation, ability to attend regular hospital visits, and voluntary consent to participate. Exclusion criteria for the study included contraindications to dapagliflozin treatment, symptoms of hypotension or a systolic blood pressure of less than 90 mm Hg, acute decompensated heart failure, and an estimated glomerular filtration rate (eGFR) below 30 mL per minute per 1.73 m^2^ of body surface area (or rapidly declining renal function).

Before starting dapagliflozin treatment for the patients, electrocardiography (ECG) and echocardiography assessments were performed as part of the comprehensive cardiac evaluation. Routine blood tests were conducted. Six months after the initiation of the dapagliflozin treatment, control ECG and echocardiography evaluations were done, and routine blood tests were repeated. To investigate the effect of the dapagliflozin treatment on immune–inflammatory markers, echocardiography and laboratory data were compared before and after treatment.

In addition to the examination of LVEF using echocardiography, the presence of heart failure symptoms and signs, pulmonary or peripheral edema, and increased NT‐proBNP levels were sought to diagnose heart failure.

HFrEF is defined as heart failure with symptoms and signs along with a LVEF < 40%.

HFmrEF is defined as heart failure symptoms and signs with a LVEF of 40–49%.

HFpEF is defined as elevated natriuretic peptide levels and LVEF ≥ 50%, along with symptoms and signs of heart failure.

The eGFR was calculated using the MDRD 4‐variable equation (age, sex, ethnicity, and serum creatinine).

LVEF was assessed using the biplane Simpson’s method from apical 4‐ and 2‐chamber views.

SII was calculated with the following formula: SII = (P × N)/L 109/L, where P, N, and L refer to peripheral platelet (PLT), neutrophil, and lymphocyte counts, respectively.

SIRI was calculated with the following formula: SIRI = (N ×M)/L 109/L, where N, M, and L refer to peripheral neutrophil, monocyte, and lymphocyte counts, respectively.

### 2.1. Statistical Analysis

All statistical analyses were performed using the SPSS for Windows Version 15.0 software (SPSS Inc., Chicago, IL, USA). The Kolmogorov–Smirnov test was used to check for normality of distribution for continuous variables. Continuous variables were presented in mean ± standard deviation (SD), while categorical variables were presented as numbers and frequencies. Paired sample *t*‐test was used to compare continuous variables before and after dapagliflozin treatment. Categorical variables were compared using Pearson’s chi‐square test and Fisher’s exact test. A *p* value of < 0.05 was considered statistically significant.

## 3. Results

Among the 191 patients in the study, 111 (58.1%) were male. The mean age of these patients was 66.17 ± 10.7 years (minimum: 41; maximum: 91). Hypertension was seen in 168 patients (88%), diabetes mellitus in 156 patients (81.7%), coronary artery disease in 155 patients (81.2%), and Stage 3 or more chronic renal disease in 14 patients (7.3%). In the baseline ECG of the patients, 173 (90.6%) had sinus rhythm and 18 (9.4%) presented with atrial fibrillation (Table 1).

**TABLE 1:** Baseline characteristics and treatments of the patients.

Variables		Patient *n* = 191
Age, years (mean ± SD)		66.17 ± 10.7
Male gender, *n* (%)		111 (58.1)
Hypertension, *n* (%)		168 (88)
Diabetes mellitus, *n* (%)		156 (81.7)
Hyperlipidemia, *n* (%)		124 (64.9)
CAD, *n* (%)		155 (81.2)
PAD, *n* (%)		3 (1.6)
CKD, *n* (%)		14 (7.3)
CVD, *n* (%)		3 (1.6)
COPD, *n* (%)		6 (3.1)
ECG rhythm	SR	173 (90.6)
	AF	18 (9.4)
Treatment		
ACE‐I/ARB, *n* (%)		130 (68.1)
ARNI, *n* (%)		14 (7.3)
Beta‐blocker, *n* (%)		147 (77)
MRA, *n* (%)		58 (30.4)
Digoxin, *n* (%)		5 (2.6)
Ivabradine, *n* (%)		19 (9.9)
Loop diuretics, *n* (%)		74 (38.7)
Thiazide diuretics, *n* (%)		24 (12.6)
Antiplatelet, *n* (%)		149 (78)
Oral anticoagulant, *n* (%)		38 (19.9)
Metformin, *n* (%)		86 (45)
GLP‐1 analogs, *n* (%)		6 (3.1)
Insulin, *n* (%)		64 (33.5)

*Note: n* = number of patients.

Abbreviations: ACE‐I = angiotensin‐converting enzyme inhibitors, AF = atrial fibrillation, ARB = angiotensin receptor blockers, ARNI = angiotensin receptor–neprilysin inhibitor, CAD = coronary artery disease, CKD = chronic kidney disease, COPD = chronic obstructive pulmonary disease, CVD = cerebrovascular disease, ECG = electrocardiography, GLP = glucagon‐like peptide, MRA = mineralocorticoid receptor antagonists, PAD = peripheral artery disease, SD = standard deviation, SR = sinus rhythm.

A total of 130 patients (68%) were taking a renin–angiotensin system (RAS) blocker, 14 (7.3%) an angiotensin receptor–neprilysin inhibitor (ARNI), 147 (77%) a beta blocker, and 58 (30.4%) a mineralocorticoid receptor antagonist (MRA). In addition, 86 patients with diabetes (45%) were prescribed metformin, 6 (3.1%) were prescribed glucagon‐like peptide‐1 (GLP‐1) analogs, and 64 (33.5%) were treated with insulin (Table 1).

The patients’ baseline LVEF was 45.2 ± 14.8%. The mean left ventricular diastolic diameter was 52.8 ± 6.6 mm, and the mean systolic pulmonary artery pressure was 32.2 ± 10.2 mmHg. Seventy patients (36.6%) had HFrEF, 31 (16.2%) had HFmrEF, and 90 (47.1%) had HFpEF. No significant changes in echocardiographic parameters were observed following dapagliflozin treatment (*p* > 0.05).

In the laboratory parameters of the patients before treatment and 6 months after the treatment initiation, no significant change was observed in the CRP value (17.9 ± 21.5 vs. 20.1 ± 36.1, *p* = 0.679), while a significant decrease was observed in the SII and SIRI with treatment (1357.4 ± 1404.3 vs. 805.8 ± 586.7, *p* < 0.001, and 3.68 ± 3.6 vs. 2.19 ± 1.7, *p* < 0.001) (Table 2).

**TABLE 2:** Pretreatment and posttreatment laboratory and echocardiographic data of patients.

Variables (mean ± SD)	Pretreatment *n* = 191	Posttreatment *n* = 191	*p* value
End‐diastolic diameter, mm	52.8 ± 6.6	51.9 ± 6.4	0.503
End‐systolic diameter, mm	36.4 ± 10.3	34.1 ± 8.8	0.292
Left atrium diameter, mm	41.1 ± 8.7	41.2 ± 8.1	0.967
sPAP, mmHg	32.2 ± 10.2	41.5 ± 13.9	0.064
LVEF, %	45.2 ± 14.8	45.2 ± 15.2	1
FBG, mg/dL	161.1 ± 63.8	154.3 ± 67.3	0.272
Urea, mg/dL	48.5 ± 30.9	49.1 ± 35.1	0.69
Creatinine, mg/dL	1.02 ± 0.3	1.06 ± 0.5	0.185
Uric acid, mg/dL	6.5 ± 1.9	5.9 ± 2.1	0.306
Sodium, mEq/L	138.1 ± 4.3	138.7 ± 4.9	0.132
Potassium, mEq/L	4.4 ± 0.6	4.4 ± 0.6	0.503
Calcium, mg/dL	9.3 ± 0.7	9.3 ± 0.8	0.937
AST, U/L	47.4 ± 106.7	32.8 ± 144.9	0.299
ALT, U/L	37.4 ± 92.4	28.3 ± 109.9	0.399
Total cholesterol, mg/dL	168.3 ± 43.7	157.4 ± 45.1	0.058
LDL, mg/dL	92.6 ± 37.8	86.5 ± 38.3	0.199
HDL, mg/Dl	42.6 ± 17.2	42.1 ± 16.3	0.771
Triglyceride, mg/dL	157.3 ± 92.1	157.2 ± 82.3	0.992
HbA1c, %	7.9 ± 2.2	7.8 ± 1.8	0.613
TSH, mUI/mL	2.4 ± 4.9	2.1 ± 2.1	0.603
T4, ng/dL	1.3 ± 0.2	1.4 ± 0.4	0.166
T3, ng/dL	2.3 ± 0.7	2.9 ± 0.9	0.127
CRP, mg/dL	17.9 ± 21.5	20.1 ± 36.1	0.679
WBC, × 10^9^/L	9.8 ± 3.4	8.1 ± 2.2	**< 0.001**
Neutrophil count, × 10^9^/L	7.2 ± 3.2	5.3 ± 1.8	**< 0.001**
Lymphocyte count, × 10^9^/L	1.8 ± 0.9	1.9 ± 0.7	**0.035**
Monocyte count, × 10^9^/L	0.69 ± 0.3	0.68 ± 0.3	0.619
Hemoglobin, g/dL	12.4 ± 2.1	12.5 ± 2.1	0.683
Hematocrit, %	38.9 ± 6.1	39.4 ± 6.1	0.198
Platelet count, × 10^9^/L	257.9 ± 85.8	251.9 ± 88.9	0.309
SII, 10^9^	1357.4 ± 1404.3	805.8 ± 586.7	**< 0.001**
SIRI, 10^9^	3.68 ± 3.6	2.19 ± 1.7	**< 0.001**

*Note: n* = number of patients; AST = aspartate aminotransferase; ALT = alanine aminotransferase. Statistically significant data (*p* values < 0.05) are presented in bold.

Abbreviations: CRP = C‐reactive protein, FBG = fasting blood glucose, HDL = high density cholesterol, LDL = low density cholesterol, LVEF = left ventricular ejection fraction, SD = standard deviation, SII = systemic immune–inflammation index, SIRI = systemic inflammation response index, sPAP = systolic pulmonary artery pressure, TSH = thyroid stimulating hormone, WBC = white blood count.

**FIGURE 1:**
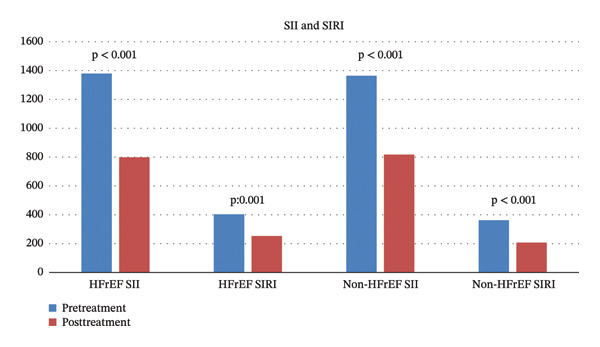
Changes in SII and SIRI with treatment according to left ventricular ejection fraction.

When changes in the SII and SIRI were analyzed according to heart failure classifications, consistent and significant reductions were achieved in both HFrEF and non‐HFrEF patients with treatment (*p* < 0.05) (Figure 1).

**FIGURE 2:**
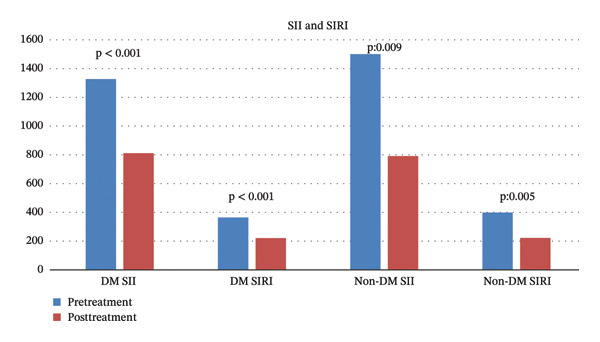
Changes in SII and SIRI with treatment according to the presence of diabetes.

Additionally, when the changes in the SII and SIRI according to the presence of diabetes were analyzed, consistent and significant reductions were achieved in both diabetic and nondiabetic patients with treatment (*p* < 0.05) (Figure 2).

## 4. Discussion

Dapagliflozin treatment significantly reduced the novel inflammatory biomarkers SII and SIRI.

Several studies have attempted to investigate the relationship between SIRI, SII, and CHF prognosis. Findings from the Medical Information Mart for Intensive Care‐III database showed that the risk of all‐cause mortality increased with rising SIRI and SII [24–26].

In CHF, activation of immune cells leads to the release of proinflammatory cytokines, activation of the complement system, production of autoantibodies, and upregulation of adhesion molecules, thereby further intensifying the inflammatory state [27]. Inflammation has been shown to be an important factor in the development and progression of HF [28–31]. When these conditions are considered together, immune responses may play an important role in the poor prognosis of CHF patients. Therefore, biomarkers reflecting immune responses may be beneficial in evaluating treatment response.

SIRI and SII are calculated using neutrophil, lymphocyte, monocyte, and PLT counts and have previously been reported to reflect the body’s systemic inflammatory response [32–34].

SII and SIRI are more valuable than evaluating a single leukocyte subtype because they reflect two aspects of the immune system. Additionally, SII can indicate the balance between PLTs and lymphocytes, coagulation function, and systemic immune response. More importantly, it remains relatively consistent despite changes in physiological conditions. Therefore, it can be considered an excellent biomarker of inflammation [23].

SII was first reported by Hu et al. These researchers reported that SII is a strong predictor of poor prognosis in patients with hepatocellular carcinoma (HCC) after liver resection [35]. Additionally, it is widely used in coronary atherosclerosis, esophageal cancer, non–small‐cell lung cancer, osteosarcoma, and other malignant tumors [36–38]. Wang Z. et al. identified SII as an important predictor of mortality in heart failure patients [23].

Possible underlying mechanisms include the following: The release of various anti‐inflammatory cytokines may trigger immune suppression, leading to lymphocyte apoptosis [39]. One study showed that HF patients with low absolute lymphocyte counts had a higher mortality risk [40]. Another study found that neutrophil counts reflect the degree of impairment of the systemic inflammatory response, and higher neutrophil counts are associated with more severe myocardial damage, impaired left ventricular function, and poorer prognosis [41]. Neutrophil infiltration was observed in the development of cardiac disease in the Physicians’ Health Study [42]. Neutrophils can cause damage to heart and kidney cells by releasing damage‐associated molecular patterns (DAMPs) from necrotic cells [43]. In our study, neutrophil counts decreased significantly with dapagliflozin treatment.

In addition to neutrophilia, thrombocytosis also increases the SII score. Mehta et al. first reported in 1979 that patients with heart failure had significantly higher PLT counts in circulation compared to the normal population [44]. Elevated PLT levels have been associated with atherosclerosis, coronary artery disease, and cerebrovascular disease, which play a role in the pathogenesis of heart failure [45, 46].

Additionally, certain physiological changes that occur during heart failure can reduce lymphocyte counts, leading to an increase in the SII. Elevated cortisol levels in heart failure reduce lymphocyte counts, while chronic inflammation increases lymphocyte apoptosis, leading to lymphopenia [47]. In line with these findings, our study observed a significant increase in lymphocyte counts following dapagliflozin treatment, though PLT counts remained unchanged.

Many studies have shown that monocytes and PLTs play an important role in the relationship between inflammation and CHF outcomes. Activated monocytes differentiate into macrophages and trigger the release of various inflammatory cytokines [48]. Monocytes mediate the inflammatory response to myocardial injury, cell apoptosis and necrosis, inflammation and immune cell activation, myocyte hypertrophy, and myocardial fibrosis [49]. An increased PLT count may result from megakaryocyte proliferation stimulated by proinflammatory cytokines and reflects inflammation activation [50].

SGLT2i are recommended to reduce hospitalizations and mortality in all heart failure patients, regardless of left ventricular function. Although the mechanism by which SGLT2i positively affect heart failure patients is not clearly known, reducing inflammation and fibrosis may be one of their most important benefits. Proposed mechanisms by which SGLT2i may exert their anti‐inflammatory effects include effects on AMPK/SIRT1/PGC‐1*α* signaling, various cytokines, and the NLRP3 inflammasome. The antioxidant effect is associated with effects on mitochondria and the transforming growth factor β and nuclear erythroid 2–related factor 2/antioxidant response element signaling pathways. Furthermore, SGLT2i exert their anti‐inflammatory and antioxidative effects by influencing metabolic parameters such as uric acid reduction, ketogenesis stimulation, body weight reduction, lipolysis, and epicardial adipose tissue [51]. Yang Z. et al. demonstrated that they reduced inflammation and fibrosis in patients with arrhythmogenic cardiomyopathy [52]. Chen et al. reported that DAPA administration protected against DM‐induced oxidative stress in the lens [53]. Wei et al. demonstrated that DAPA improved pancreatic β‐cell function in db/db mice [54]. Data from the in vivo part of our study demonstrate that treatment with DAPA lowered glucose levels and blood pressure and improved heart function concomitant with reduced inflammation and fibrosis in angiotensin II (ATII)–stressed db/db mice [55]. In our study, the new inflammation markers SII and SIRI were significantly reduced with dapagliflozin treatment.

In addition to left ventricular failure, right ventricular failure is also frequently observed in CHF patients. Studies have shown that right ventricular heart failure is the most important predictor of poor outcomes in CHF [56, 57]. In a meta‐analysis conducted by Cinar et al., it was determined that SGLT2i improved right ventricular functions in CHF patients [58].

### 4.1. Limitations

The most important limitation of our study is that it was not designed as a randomized controlled trial. Another important limitation is that, while we demonstrated a significant improvement in new inflammation markers with dapagliflozin treatment, we were unable to assess the effect of dapagliflozin on fibrosis development. Finally, the positive results of our study need to be supported by larger population studies.

## 5. Conclusion

Dapagliflozin is an important treatment that reduces hospital admissions and mortality in patients with heart failure. In our study, the SII and SIRI, which are novel inflammation parameters, decreased significantly with dapagliflozin treatment. This effect may contribute to the CV benefits associated with dapagliflozin treatment.

## Funding

The authors received no financial support for the research, authorship, and/or publication of this article.

## Ethics Statement

Local ethics committee approval was obtained for the study from Bakırcay University’s noninterventional clinical research ethics committee (decision number: 2050‐12.02.2024). The study protocol conforms to the ethical guidelines of the 1975 Declaration of Helsinki.

## Consent

Written informed consent was obtained from each patient included in the study.

## Conflicts of Interest

The authors declare no conflicts of interest.

## Data Availability

The data that support the findings of this study are available from the corresponding author upon reasonable request.
